# Combating *Klebsiella pneumoniae*: from antimicrobial resistance mechanisms to phage-based combination therapies

**DOI:** 10.3389/fcimb.2025.1691215

**Published:** 2025-11-07

**Authors:** Jiaxin Ding, Weilei Yan, Rui Zheng, Mengye Ma, Liming Jiang

**Affiliations:** 1 School of Basic Medical Sciences, Health Science Center, Ningbo University, Ningbo, China; 2 Department of Clinical Laboratory, The First People’s Hospital of Yunnan Province, Kunming, China

**Keywords:** K.pneumoniae, phage, antimicrobial resistance mechanisms, phage-based combination therapy, clinical translation

## Abstract

*Klebsiella pneumoniae* (*K. pneumoniae*) is a central pathogen in both nosocomial and community-acquired infections worldwide, capable of causing pneumonia, urinary tract infections (UTIs) and bacteremia. In recent years, the spread of multi-drug resistant (MDR) bacterial pathogens has become a major public health challenge. Traditional antibiotics, which are increasingly ineffective due to escalating resistance, significant adverse effects, and limited therapeutic efficacy, underscore the urgent need for novel strategies. The primary antimicrobial resistance mechanisms of *K. pneumoniae* currently include alterations of drug target sites, modified enzyme-mediated antibiotic inactivation, permeability barriers to antimicrobial agents, active efflux systems, synergistic resistance mechanisms involving biofilm-persisters-quorum sensing (QS) and heteroresistance. While phage therapy offers precise targeting of pathogenic bacteria, its standalone use is hampered by obstacles such as the rapid evolution of bacterial resistance and narrow host ranges. Accordingly, combinatorial phage therapy has emerged as a key research focus. In this review, we not only summarize the multidimensional antimicrobial resistance mechanisms of *K. pneumoniae* and the principles of synergistic phage strategies but also evaluate the potential for clinical translation and current challenges, providing a theoretical framework for the precise treatment of multidrug-resistant *K. pneumoniae* (MDRKP) infections, so as to promote the clinical application of phage-based combination therapy in the post-antibiotic era. Beyond summarizing recent advances, this work also provides a unique translational perspective by critically evaluating the synergy, clinical applicability, and challenges of combinatorial phage approaches—including phage-antibiotic, phage-AMP, and phage-nanocarrier therapies—against MDRKP, filling a critical gap in existing reviews.

## 1 Introduction

*K. pneumoniae*, a Gram-negative opportunistic pathogen, is classified under the genus *Klebsiella* in the Enterobacteriaceae family. As a facultative anaerobe, *K. pneumoniae* exhibits remarkable adaptability by thriving in both aerobic and anaerobic environments. This bacterium is ubiquitously distributed across natural habitats, including water, soil, and plant surfaces, as well as within the human body, where its primary colonization sites are the gastrointestinal tract and the oropharynx; the skin may occasionally harbor this bacterium (Martin and Bachman, 2018). This strain has significant clinical pathogenic potential and can cause community-acquired infections as well as nosocomial infections (Pu et al., 2023), such as pneumonia, UTIs and bacteremia (Paczosa and Mecsas, 2016). Notably, compared to infections at other sites, bloodstream infections caused by this pathogen demonstrate a case fatality rate ranging from 20% to 33% (Seo et al., 2020). Epidemiological data show *K. pneumoniae* infections spread across Asia, Africa, Europe, and the Americas, hitting resource-limited, high-density areas hardest. From a “One Health” lens (linking human, animal, environmental health), its cross-species transmission amplifies threats. Asia is a hotspot: MDR-hypervirulent strains exist in India, Pakistan, Vietnam (Silvester et al., 2022), 99.4% of Indian isolates carry *bla_OXA_* variants (Jean et al., 2024). Egypt has horse-derived *K. pneumoniae* (100% cefotaxime-resistant) with biofilm genes (Arafa and Kandil, 2024). Egyptian bovine mastitis cases (28%) have MDRKP (100% beta-lactam resistance) (Shehata et al., 2024), proving livestock as reservoirs. Tackling it needs One Health strategies. In Asia, particularly in India and Southeast Asian countries, *K. pneumoniae* is the major pathogen of bloodstream infections, and the coexistence of MDR and highly virulent strains is gaining popularity (Wyres et al., 2020; Raj et al., 2022). In Africa, 42% of clinical *K. pneumoniae* isolates in Kenya were MDR, carrying multiple resistance genes (e.g., *bla_NDM-1_* and *bla_OXA-181_*) and high virulence genes (e.g., *rmpA* and *magA*) (Muraya et al., 2022). Studies conducted in Europe and the Americas have demonstrated that nosocomial transmission and clonal spread of *K. pneumoniae* are the main causes of infection outbreaks, particularly in intensive care units (ICUs) and among long-term hospitalized patients (da Silva et al., 2021; Pei et al., 2022). Moreover, studies in Turkey have shown that *K. pneumoniae* exhibits a high carbapenem resistance rate of 49.7%, with *bla_OXA-48_* being the most prevalent resistance gene (Süzük Yıldız et al., 2021).

In recent decades, the global prevalence of MDRKP has risen dramatically, particularly with carbapenem-resistant *K. pneumoniae* (CRKP) showing a progressive annual increase in detection rates. CRKP has been designated as a critical priority pathogen in the World Health Organization’s (WHO) 2024 list of “Antimicrobial-Resistant Bacteria Posing the Greatest Threat to Human Health.” Surveillance data reveal concerning trends: The European Antimicrobial Resistance Surveillance Network (EARS-Net) 2023 report indicates CRKP bloodstream infection incidence in EU countries reached 3.97 cases per 100,000 population in 2023 - a 57.5% increase from 2019 and exceeding the 2030 target (2.39/100,000). China’s CHINET surveillance network reported a 10.8% CRKP detection rate in 2023, representing a 1.6-fold increase since 2014 (Guo et al., 2024). The development of antimicrobial resistance in *K. pneumoniae* stems from synergistic multidimensional molecular mechanisms, including drug target modification, enzyme-mediated antibiotic inactivation, antimicrobial permeability barriers, overexpression of efflux pumps, as well as complex mechanisms involving biofilm formation, persisters, and QS regulation. The escalating antibiotic misuse and transmission of CRKP have contributed to rising antimicrobial resistance, resulting in a consistent annual increase in CRKP detection rates. For decades, polymyxins, tigecycline, and ceftazidime/avibactam (CAZ/AVI, a novel β-lactam/β-lactamase inhibitor combination) have remained the primary therapeutic options for CRKP infections (Watkins and Deresinski, 2015; Shankar et al., 2017; Petrosillo et al., 2019). However, these antibiotics carry risks of toxicity and have limited efficacy. As a result, there is an urgent need to develop new alternative antibiotic therapies to address the evolving problem of MDRKP.

Among current alternative therapies, phage therapy has shown remarkable efficacy (Kinanti. et al., 2024). Phages are viruses that specifically infect and replicate within bacterial cells, exhibiting high host specificity (Gradisteanu Pircalabioru et al., 2021). Their mechanism involves invading bacterial cells, undergoing replication, and ultimately lysing the cell wall to induce bacterial death. Phages are primarily categorized into two classes: lytic and lysogenic. Lytic phages undergo rapid replication following infection, culminating in host cell lysis and the subsequent release of progeny virions, rendering them particularly effective for prompt eradication of bacterial infections. In contrast, lysogenic phages integrate their genetic material into the bacterial genome without immediate bactericidal effects, initiating the lytic cycle only under specific conditions. Comparatively, temperate phages exhibit significantly lower bactericidal efficacy and are generally not preferred for therapeutic applications. These fundamental characteristics of phages endow them with distinctive potential in combating drug-resistant bacteria. Their exceptional specificity, targeting only particular bacterial species without causing harm to human cells, holds particular significance in modern medicine, as it ensures minimal disruption to the host’s microbiome during antibacterial treatment (Liu et al., 2021b). Besides, the vast abundance and diversity of naturally occurring phages offer substantial potential for their development as antibiotic alternatives in disease treatment. Building upon conventional phage therapy, phage cocktail combination therapy demonstrates multi-level synergistic advantages against MDRKP. This strategy combines phages with antibiotics, antimicrobial peptides, photosensitizers, and other mechanistically distinct components to produce synergistic effects such as “lysis-permeabilization” and “membrane disruption-lysis,” significantly enhancing bactericidal efficiency while reducing drug resistance risk (Mirski et al., 2019; Wang et al., 2021a, b; Petrosino et al., 2023; Tyagi et al., 2024). The multi-target intervention not only overcomes intrinsic bacterial resistance (e.g., increased antibiotic susceptibility in phage-resistant strains) but also effectively minimizes bacterial escape and improves targeting through techniques like phage cocktails and nanocarrier delivery systems (Rohde et al., 2018; Wunderink, 2019; Qamar et al., 2022; Wang et al., 2024). Furthermore, the combined application with probiotics and quorum sensing inhibitors (QSIs) achieves comprehensive outcomes including pathogen eradication, microbiome restoration, and bacterial defense attenuation, providing a breakthrough solution for clinical MDRKP infections while ensuring therapeutic safety (Liu et al., 2021a; Martínez et al., 2023).

This review systematically summarizes the most recent advancements in *K. pneumoniae* research, with particular emphasis on antimicrobial resistance mechanisms and phage cocktail-based combination therapeutic approaches. Additionally, it identifies priority research areas and key scientific questions that should be addressed in future studies on phage therapy for *K. pneumoniae* infections.

While several recent reviews have summarized phage therapy against *K. pneumoniae*, this review uniquely provides a systematic integration of multidimensional antimicrobial resistance mechanisms with advanced phage-based combination strategies, including phage-antibiotic, phage-AMP, phage-nanocarrier, and phage-QSI synergies. Furthermore, we critically evaluate the clinical translatability and persistent challenges of these approaches, offering a forward-looking perspective tailored for the post-antibiotic era. This comprehensive and mechanistic focus distinguishes our work and provides a foundational framework for developing precision therapies against MDRKP.”

## 2 Mechanisms of antimicrobial resistance in *K. pneumoniae*

**Figure 1:**
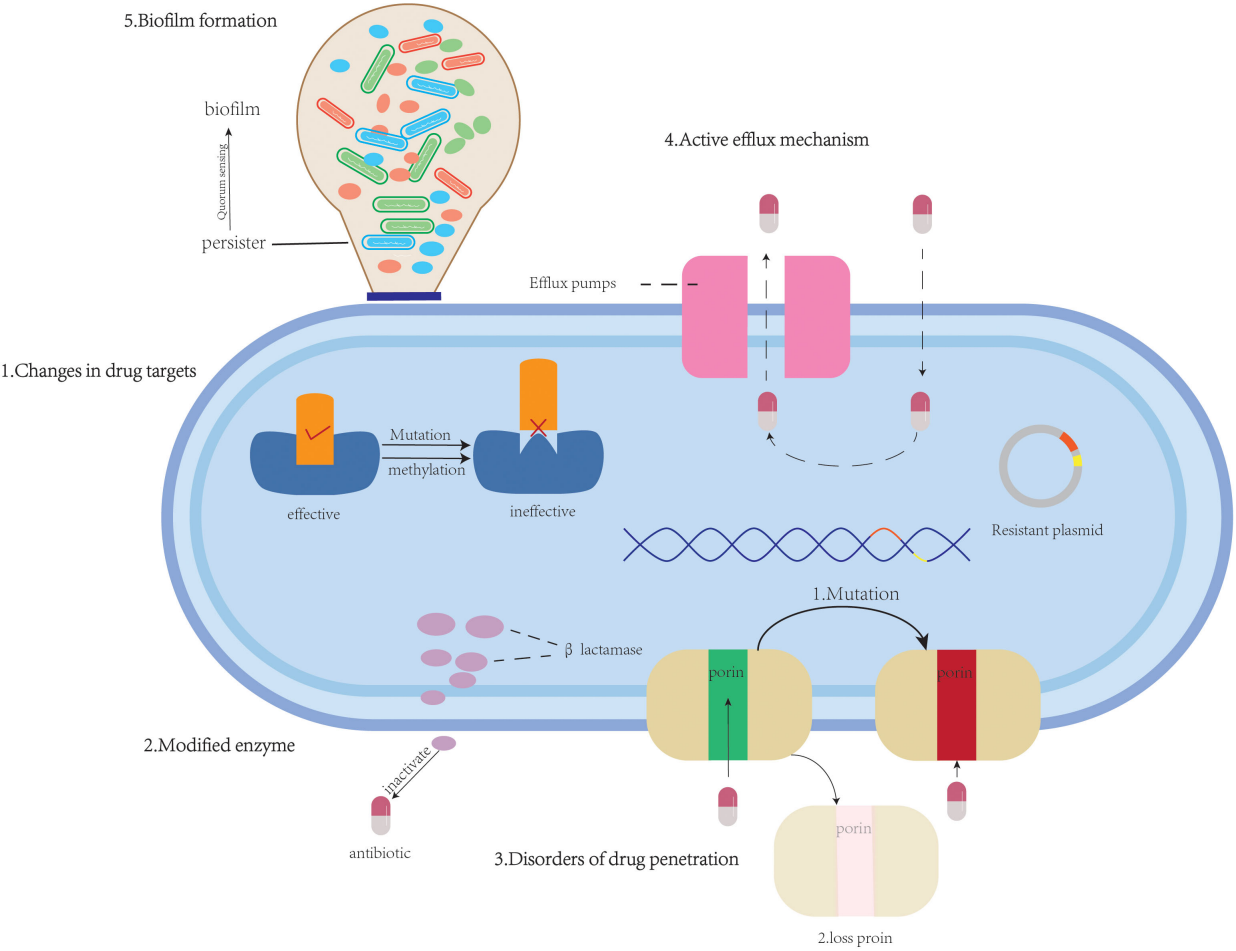
An overview of antimicrobial resistance mechanisms: (1) alterations of drug target sites (β-lactams, tetracyclines, aminoglycosides, quinolones, polymyxins); (2) modified enzyme (β-lactams, aminoglycosides, chloramphenicols, macrolide); (3) permeability barriers to antimicrobial agents (β-lactams); (4) active efflux systems (β-lactams, quinolones, tetracyclines, macrolides, chloramphenicols, aminoglycosides, dyes, and surfactants);(5) biofilm formation (β-lactams, aminoglycosides).

The development of antimicrobial resistance in *K.pneumoniae* arises from the synergistic interplay of multidimensional molecular mechanisms. These include: (1) alterations of drug target sites (e.g., mutations in PBPs (Jiang et al., 2020), ribosomal methylation (Liou et al., 2006), mutations in DNA gyrase/topoisomerase (Park et al., 2017; Onishi et al., 2022)and modification of lipid A (McConville et al., 2020)); (2) modified enzyme-mediated antibiotic inactivation (e.g., β-lactamases); (3) permeability barriers [e.g., capsular polysaccharide (CPS) thickening (Wang et al., 2025), Lipopolysaccharide (LPS) modifications (Wareth and Neubauer, 2021) and porin mutations/deletions (Khalid et al., 2020; Li et al., 2022)]; (4) active efflux systems (e.g., AcrAB-TolC); (5) synergistic resistance mechanisms involving biofilm-persisters-QS; and (6) heteroresistance. These mechanisms frequently evolve dynamically through horizontal gene transfer (HGT) or mutation accumulation, forming intricate resistance networks. They are primarily responsible for the dramatic global increase in MDRKP prevalence and the emergence of strains exhibiting MDR, extensive drug resistance (XDR), and even pan-drug resistance (PDR), posing significant challenges to clinical treatment. Meanwhile, with the widespread use and misuse of antibiotics, the issue of antimicrobial resistance in *K. pneumoniae* has become increasingly severe. In particular, the spread of CRKP has emerged as a global concern. Therefore, a deeper understanding of the molecular basis of its resistance mechanisms is crucial for developing novel anti-infective strategies. This section aims to systematically review recent advances in the antimicrobial resistance mechanisms of *K. pneumoniae*, integrating molecular insights to provide a theoretical foundation for mechanism-driven antimicrobial approaches. The various mechanisms by which *K. pneumoniae* develops resistance to antibiotics are portrayed in **Figure 1**.

### 2.1 Alterations of drug target sites

#### 2.1.1 Mutation of penicillin-binding proteins

PBPs are essential enzymes for bacterial cell wall synthesis, catalyzing peptidoglycan cross-linking reactions and serving as primary targets for β-lactam antibiotics. These antibiotics exert their bactericidal effects by enhancing binding affinity to PBPs, thus inhibiting bacterial growth. For instance, meropenem inhibits PBP activity to disrupt cell wall biosynthesis (Mostafa Abdalhamed et al., 2021). Nevertheless, bacteria develop resistance through PBP mutations or modifications that reduce antibiotic-target affinity. Jiang et al. (2020) experimentally demonstrated that *K. pneumoniae* acquires meropenem resistance through PBP mutations that decrease drug-binding affinity, thus reducing susceptibility to β-lactam antibiotics and enhancing *K. pneumoniae* ‘s overall resistance.

It’s worth noting that PBP3 (encoded by the *ftsI* gene) serves as the primary target of CAZ. Mutations in PBP3 can reduce CAZ-PBP3 binding affinity, consequently leading to CAZ-AVI resistance. The L367Q mutation (leucine-to-glutamine substitution in PBP3) observed in strain 84082-IR likely alters PBP3’s active site, diminishing its CAZ-binding capacity and significantly increasing CAZ-AVI resistance (Guo et al., 2021). Other studies revealed that a four-amino-acid (T-I-P-Y) insertion in PBP3 can markedly enhance CAZ-AVI resistance by modifying PBP3’s conformation and reducing its binding affinity for β-lactam antibiotics (Zhang et al., 2017).

#### 2.1.2 Modification of ribosomal target site

Ribosomal target modifications contribute significantly to tigecycline resistance in *K. pneumoniae*, primarily through mutations in the S10 protein. A key example is the Val57Leu mutation within a conserved region near the tigecycline binding site on the 30S ribosomal subunit. This mutation is thought to alter ribosome conformation or hinder antibiotic binding, thereby reducing drug efficacy (He et al., 2018). Unlike mechanisms such as efflux pump overexpression, this approach directly modifies the ribosome to impede antibiotic action, highlighting a novel resistance pathway specific to tigecycline (Zhang et al., 2021).

Another major mechanism is ribosomal methylation mediated by enzymes such as ArmA, which methylates the G1405 position of 16S rRNA. This modification decreases aminoglycoside binding affinity through structural interference and electrostatic effects, conferring broad resistance to most aminoglycosides except streptomycin (Liou et al., 2006). The ArmA gene is often plasmid-borne or located on mobile genetic elements, facilitating its dissemination among clinical isolates of *K. pneumoniae* and other Gram-negative bacteria (Galimand et al., 2003, 2005).

In contrast to the aforementioned strategy whereby bacteria develop resistance by modifying ribosomal target sites, the design of novel antimicrobial agents targeting highly conserved ribosomal sites is considered a promising approach to overcome resistance. Studies have demonstrated that the binding site of the optimized proline-rich antimicrobial peptide (PrAMP) Onc112 on the 23S rRNA is highly conserved among various pathogens, including *K. pneumoniae*. Onc112 exhibits exceptionally high affinity (Kd=77 nM) for the ribosomes of *K. pneumoniae*. However, its effective antibacterial activity (MIC=2 mg/L) is still constrained by factors such as bacterial uptake efficiency. This finding underscores the value of Onc112 as a lead structure. Developing drugs based on this scaffold that target conserved ribosomal sites may help circumvent resistance arising from target site mutations, thereby offering a new direction for addressing the drug resistance challenges posed by *K. pneumoniae* (Kolano et al., 2020).

#### 2.1.3 Mutations in DNA gyrase and topoisomerase

DNA gyrase (encoded by *gyrA* and *gyrB*) and topoisomerase IV (encoded by *parC* and *parE*) are essential enzymes for DNA replication and transcription in *K. pneumoniae*, catalyzing DNA strand breakage and rejoining. Quinolone antibiotics exert their antibacterial effects by inhibiting the activity of these enzymes, thus blocking DNA replication. In recent years, quinolone resistance in *K. pneumoniae* has increased, with mutations in *gyrA* (DNA gyrase) and *parC* (topoisomerase IV) identified as the primary resistance mechanism. These mutations occur within the quinolone resistance-determining regions (QRDRs), particularly *gyrA*-Ser83Ile and *parC*-Ser80Ile substitutions, which induce conformational changes that markedly reduce quinolone binding affinity (Park et al., 2017; Onishi et al., 2022). A study on ESBL-producing *K.pneumoniae* isolates from Indonesia demonstrated that strains with QRDR mutations exhibited high resistance rates of 82.9% to ciprofloxacin (CPFX) and 69.5% to levofloxacin (LVFX), with these mutations showing significant correlation with quinolone resistance. Additionally, *gyrA*-Ser83Phe and *gyrA*-Asp87Ala mutations were associated with resistance to nalidixic acid (NA) and LVFX. Although plasmid-mediated quinolone resistance (PMQR) genes (e.g., *qnr*, *aac(6’)-Ib-cr*) contribute to resistance in some strains, QRDR mutations exert a far greater impact. Strains carrying both QRDR mutations and PMQR genes demonstrate significantly higher resistance than those with PMQR alone, though QRDR mutations alone suffice for high-level resistance (Zhan et al., 2021). Conjugation experiments confirmed that the ESBL gene *bla_CTX-M-15_* and PMQR gene *aac(6’)-Ib-cr* can be horizontally transmitted via plasmids, exacerbating resistance dissemination—particularly in ICUs (Bado et al., 2016). These findings underscore the dominance of QRDR mutations in quinolone resistance, urging judicious clinical use of quinolones, especially in high-resistance settings, to curb further spread.

#### 2.1.4 Modification of lipid A

Lipid A, a key component of lipopolysaccharide in Gram-negative bacteria, serves as the primary target of polymyxins and contributes significantly to polymyxin resistance in *K. pneumoniae*. This resistance is mainly mediated through enzymatic additions of 4-amino-4-deoxy-L-arabinose (L-Ara4N) by ArnT and phosphoethanolamine (pEtN) by EptA. These modifications increase the positive charge of the bacterial membrane, electrostatically repelling cationic polymyxins (McConville et al., 2020).

Two-component systems (TCSs) play a central role in regulating lipid A modifications. CrrA/B, PmrA/B, and PhoP/Q are key TCSs involved. CrrA/B activation—often through CrrB mutations (e.g., L87V, L94M, P151S)—upregulates both the *arnBCADTEF* operon (ArnT) and *pmrC* (EptA), leading to dual modifications and increased polymyxin MIC (Macesic et al., 2020). PmrA/B, regulated by CrrB, similarly enhances these modifications. PhoP/Q is negatively regulated by MgrB; MgrB inactivation (e.g., via IS5-like insertions) relieves this repression, upregulating lipid A modification genes (Cannatelli et al., 2013; McConville et al., 2020).

Synergistic mutations (e.g., *crrB* and *mgrB*) further enhance lipid A modifications via multiple pathways. For instance, the *crrB151_ΔmgrB* mutant shows upregulation of both *arnBCADTEF* and *pmrC*, resulting in higher MICs. Additionally, TCSs interact with metabolic pathways such as the pentose phosphate pathway (PPP), which supplies precursors for L-Ara4N and pEtN synthesis (McConville et al., 2020). Together, these regulatory networks significantly promote polymyxin resistance and represent potential targets for novel inhibitors.

### 2.2 Modified enzyme

Modified enzymes are a class of bacterial enzymes produced by resistant strains that can degrade or inactivate antimicrobial agents. As one of the most critical mechanisms underlying MDR, these enzymes interfere with the efficacy of drugs by hydrolyzing or modifying them before they can act on bacterial targets. In *K. pneumoniae*, the production of modified enzymes is predominantly mediated by mobile genetic elements (MGEs), including plasmids, insertion sequences, transposons, and integrons. These elements facilitate the enhancement and dissemination of resistance through HGT, genetic mutations, or recombination, serving as key contributors to the emergence of clinically relevant MDR strains. For example, *bla_OXA-48_* is predominantly harbored by IncL/M-type plasmids, which display inter-strain transferability that significantly facilitates the dissemination of antibiotic resistance (Nicitra et al., 2025). DHA-1-overexpressing mutants exhibit markedly enhanced resistance to both CAZ and the novel β-lactam/β-lactamase inhibitor combination ceftolozane/tazobactam (Barceló et al., 2024). Current research advances in resistance mechanisms mediated by β-lactamases and aminoglycoside-modifying enzymes (AMEs) in *K. pneumoniae* are summarized in **Table 1**.

**Table 1:** A Summary of resistance mechanisms mediated by classical enzyme types of β-lactamases and AMEs in *K. pneumoniae*.

Enzyme name		Representative enzyme type	Resistance mechanism	Refs
β-lactamases	extended-spectrum beta-lactamases (ESBLs)	CTX-M-15,SHV-12,TEM-1	Hydrolysis of β-lactam antibiotics (penicillins, cephalosporins, and monobactams)	(Carvalho et al., 2021; Karunasagar et al., 2021; Ghenea et al., 2022)
	K.pneumoniae carbapenemase (KPC)	KPC-2,KPC-3	Hydrolytic activity against carbapenems (imipenem, meropenem), penicillins (ampicillin), and cephalosporins	(Tooke et al., 2023; Tang et al., 2024; Zhang et al., 2024)
	AmpC β-lactamases	CMY-2,DHA-1	Hydrolysis of β-lactam antibiotics (cephalosporins and penicillins)	(Oliveira et al., 2022; Barceló et al., 2024)
	Metallo-β-lactamases (MBLs)	NDM-1,VIM-2,IMP-1	Zinc ions present in the active site catalyze β-lactam ring hydrolysis, resulting in the inactivation of carbapenems (imipenem and meropenem), cephalosporins, and monobactams.	(Ejikeugwu et al., 2021; Kot et al., 2023; Wei et al., 2023)
	OXA-type carbapenemases	OXA-48	Hydrolysis of carbapenem antibiotics (meropenem and imipenem), broad-spectrum cephalosporins, and non-β-lactam antibiotics (aminoglycosides)	(Villacís et al., 2020; Sharma et al., 2022; Nicitra et al., 2025)
AMEs	Acetyltransferases (AACs)	AAC(6′)-Ib	Acetylation-mediated modification of the 6′-hydroxyl group in aminoglycoside antibiotics (tobramycin, amikacin, and netilmicin)	(El-Gebaly et al., 2021; Foudraine et al., 2021)
	Phosphotransferases (APHs)	APH(3’)-Ib	Phosphorylation at the 3’-hydroxyl position of aminoglycoside antibiotics (kanamycin and neomycin)	(Pragasam et al., 2020; Mutuku et al., 2022; Otun et al., 2024)
	Nucleotidyltransferases (ANT)	ANT(2″)-I	Enzymatic phosphorylation or adenylation modifications of aminoglycoside antibiotics (gentamicin and tobramycin)	(Foudraine et al., 2021, 2022)

### 2.3 Permeability barriers to antimicrobial agents

#### 2.3.1 CPS

The CPS of *K. pneumoniae* forms a physical barrier that impedes antimicrobial agents from accessing the bacterial surface, thus reducing drug permeability. Shweta Singh et al. experimentally demonstrated that these polysaccharides enhance capsular retention through interactions with LPS O-antigens. It should be noted that *WaaL* gene inactivation significantly diminishes capsular retention in *K. pneumoniae*, resulting in heightened susceptibility to antimicrobial agents (Singh et al., 2022).

Additionally, studies indicate that CPS thickness and structure directly influence *K. pneumoniae*’s susceptibility to complement-mediated killing. Wang et al. (2025) demonstrated that isoferulic acid (IFA), a natural compound inhibiting CPS synthesis, significantly reduces capsule thickness in *K. pneumoniae*. Accordingly, this reduction enhances bacterial vulnerability to complement-mediated killing, providing inverse evidence that increased CPS production confers greater resistance to antibiotic clearance.

#### 2.3.2 LPS

The LPS of *K. pneumoniae* serves as a crucial virulence factor whose modifications can alter membrane permeability, thereby restricting antimicrobial penetration. This modification mechanism represents a significant pathway for *K. pneumoniae* to develop antibiotic resistance. For example, O-antigen modifications in *K. pneumoniae* modify LPS charge and structure, which reduces β-lactam drug interactions with the membrane and therefore limits transmembrane drug transport. This barrier prevents these antibiotics from entering bacterial cells to exert bactericidal effects. Particularly prominent in clinically isolated MDRKP strains, this mechanism substantially exacerbates treatment challenges (Wareth and Neubauer, 2021).

Moreover, the upregulation of LPS biosynthesis serves as a critical bacterial strategy against antibiotics. Key enzymes in LPS biosynthesis (e.g., LpxC) are highly conserved among most gram-negative bacteria, with their activity directly influencing both LPS production and bacterial viability. For instance, small-molecule inhibitors targeting LpxC demonstrate potent antibacterial activity against *K. pneumoniae in vitro*, indicating that suppressing LPS biosynthesis can counteract bacterial resistance (Romano and Hung, 2023).

#### 2.3.3 Porins

Porins are β-barrel transmembrane proteins that facilitate the diffusion of small molecules, including antibiotics. In *K. pneumoniae*, OmpK35 and OmpK36 are major porins. Strains deficient in both show markedly increased antibiotic resistance (Khalid et al., 2020).

Porin deficiency or dysfunction reduces membrane permeability and antibiotic influx, contributing notably to MDR. Mutations or deletions in *ompK35* or *ompK36* lead to reduced expression or defective porins, decreasing intracellular antibiotic accumulation and raising MICs. For example, CAZ/AVI-resistant strains often exhibit OmpK35/36 deficiencies, such as a premature stop codon in OmpK35 or an insertion in OmpK36, impairing antibiotic penetration (Li et al., 2022).

### 2.4 Active efflux systems

Efflux pumps are membrane transporters that expel antibiotics, reducing intracellular concentrations and conferring resistance. In *K. pneumoniae*, the dominant systems are AcrAB-TolC and OqxAB (RND family), which have broad substrate specificity. These systems contribute significantly to resistance against diverse antimicrobials (Al-Lami et al., 2022). Major efflux families include RND, SMR, MFS, ABC, and MATE. All documented resistance genes or systems within the RND family, along with their corresponding antimicrobial agents, are described in **Table 2**.

**Table 2:** Resistance genes/systems within the RND family along with corresponding antimicrobial agents.

Gene/system name	Antimicrobial agents	Refs
AcrAB-TolC	β-lactams, quinolones, tetracyclines, macrolides, chloramphenicol, aminoglycosides, dyes (acriflavine, ethidium Br, rhodamine 6G), surfactants (sodium dodecyl sulfate (SDS), tetraphenylphosphonium Cl (TPP Cl)), Na cholate	(Li et al., 2020; Ni et al., 2020; Lv et al., 2021; Zhang et al., 2023)
OqxAB	β-lactams, aminoglycosides, tetracyclines, fluoroquinolones, chloramphenicol, nitrofurans, dyes (acriflavine, ethidium Br, rhodamine 6G), surfactants (SDS, TPP Cl), Na cholate	(Ni et al., 2020; Razavi et al., 2020; Zhou et al., 2021; Hussein et al., 2024)
tmexCD1-toprJ1	Tetracyclines, quinolones, cephalosporins, and aminoglycosides	(Lv et al., 2020; Liu et al., 2022)
kexEF, EefAB	β-lactams, macrolides, tetracyclines, quinolones, dyes (acriflavine, ethidium Br, rhodamine 6G), surfactants (SDS, TPP Cl), Na cholate	(Ni et al., 2020)
kexC	β-lactams, macrolides, and dyes (acriflavine, ethidium Br)	(Ni et al., 2020)
kexD	macrolides, dyes (acriflavine, ethidium Br)	(Ni et al., 2020)
kexVWX	dyes (acriflavine, ethidium Br) surfactants (SDS)	(Ni et al., 2020)
kexJK	surfactants (SDS)	(Ni et al., 2020)
kexSR, kexTU	dyes (acriflavine, ethidium Br)	(Ni et al., 2020)

### 2.5 Synergistic resistance mechanisms involving biofilm-persisters-QS

Biofilms are structured microbial communities formed by aggregated microorganisms (e.g., bacteria, fungi, etc.) that adhere to biotic or abiotic surfaces and become embedded within a self-secreted extracellular polymeric substance (EPS) matrix. This matrix primarily consists of extracellular polysaccharides, proteins, lipids, and DNA, forming a physical barrier that protects the enclosed microorganisms from external threats. *K. pneumoniae* frequently forms biofilms on medical devices and surfaces, utilizing this protective mechanism to shield bacterial cells from adverse host conditions (e.g., hypoxia and nutrient deprivation) and antimicrobial agents (Perez, 2019). The antimicrobial resistance of *K. pneumoniae* in the biofilm state is 10 to 1000 times higher than in the planktonic state (Mohamed et al., 2018), which poses significant challenges for diagnosis and treatment.

The mechanisms underlying enhanced bacterial drug resistance following biofilm formation are complex and multifaceted. Research indicates that EPS, as the core component of biofilms, employs its polysaccharide network to restrict drug diffusion through dual mechanisms: physical barrier effects and charge-mediated interactions (Shadkam et al., 2021). Firstly, the dense mesh structure formed by EPS can effectively block large molecule antibiotics (e.g., β-lactams and carbapenems) from entering into the interior of the biofilm, resulting in the concentration of the drug inside the membrane being significantly lower than the bactericidal threshold. Shadkam et al. reported that 75% of *K. pneumoniae* clinical isolates exhibited biofilm-forming capacity, with biofilm-producing strains demonstrating significantly higher antibiotic resistance than non-biofilm formers (p<0.05). It’s worth noting that these strains showed 10-20-fold increased resistance to cephalosporins and carbapenems—a phenomenon likely attributable to the physical barrier function of EPS. Secondly, the negatively charged EPS can electrostatically interact with positively charged antibiotics (e.g., aminoglycosides), which further diminishes drug efficacy. In Shadkam et al.’s study, 98% of tested isolates carried the *luxS* gene (a Type II QS regulatory system), whose overexpression may enhance the charge barrier effect by promoting EPS synthesis, consequently exacerbating drug penetration resistance and amplifying antimicrobial tolerance. What’s more, strains isolated from hydrophobic medical device surfaces (e.g., PVC urinary catheters) exhibited stronger adhesive capabilities, suggesting that material properties may indirectly augment drug resistance by facilitating biofilm densification.

Besides, flagellar proteins (FliG, FlgE, FlgM) and pilus proteins (StbA, TraC, FimC) of *K. pneumoniae* modulate bacterial motility and biofilm formation, thereby influencing drug resistance. Sharma et al. (2019) demonstrated through proteomic analysis that in meropenem-induced CRKP clinical strains, downregulation of these structural proteins reduced bacterial motility and promoted biofilm-like state formation—a critical factor enabling bacterial tolerance to high-dose antibiotics. These changes may ultimately enhance meropenem resistance. Bacteria within biofilms predominantly exist in a metabolically dormant and slow-growing state, rendering them largely insensitive to antimicrobial agents. It has been shown that a fraction of bacteria within the biofilm are transformed into persisters, cells that significantly reduce their metabolic activity by entering a dormant state and thus show a high degree of resistance to antimicrobial drugs. Persisters differ from conventional drug-resistant strains; they represent non-replicating or slow-growing bacterial populations that survive antibiotic pressure through metabolic quiescence. These cells possess remarkable antibiotic tolerance, enabling survival during antimicrobial exposure while retaining the capacity to resume proliferation upon antibiotic removal, remaining susceptible to the original drugs. This adaptive strategy constitutes a key contributor to chronic infections and therapeutic failures. Pourmehdiabadi et al. (2024) documented that *K. pneumoniae* within biofilms could survive high-concentration colistin exposure by entering a metabolically dormant state. The activation of toxin-antitoxin (TA) systems (e.g., HipA/HipB and MazE/MazF) facilitates persister formation and maintenance by modulating cellular growth and metabolism. Specifically, the MazF toxin induces bacterial dormancy by suppressing the synthesis of DNA, RNA, and proteins (Abokhalil et al., 2020).

QS is an intercellular communication mechanism whereby bacteria secrete and detect specific chemical signaling molecules (e.g., autoinducers) to monitor population density in real time. When these molecules reach a critical threshold concentration, they synchronously regulate gene expression. This system enables bacterial populations to coordinate collective behaviors (e.g., virulence factor secretion, biofilm formation, and antibiotic tolerance), transitioning from individual actions to cooperative group dynamics, thus enhancing environmental adaptability and survival competitiveness (Hetta et al., 2024).

Autoinducers are primarily classified into two major categories, each defined by distinct systems: Type I QS and Type II QS. In Type I QS systems, the signaling molecules serving as autoinducers are derivatives of N-acyl homoserine lactones (AHLs), while the signaling molecule in Type II QS systems is designated as autoinducer-2 (AI-2) (Li and Nair, 2012; Pereira et al., 2013).

Type I QS is a highly specific system for intraspecies communication, whereas type II QS is considered to facilitate interspecies interactions, enabling bacteria to respond not only to their AI-2 but also to AI-2 produced by other species. Although AHLs have not been demonstrated in *K. pneumoniae* isolates, exogenous AHLs can attenuate biofilm formation capacity in this pathogen. Experimental evidence shows that exogenous AHLs (e.g., C6-HSL and 3-oxo-C6-HSL) significantly reduce biofilm biomass in moderate and strong biofilm-forming strains, suggesting that QS signaling may negatively regulate biofilm formation while enhancing *K. pneumoniae*’s drug resistance (Wusiman et al., 2024).

LuxS serves as the key enzyme in Type II QS systems, catalyzing the production of DPD (4,5-dihydroxy-2,3-pentanedione), the precursor molecule that spontaneously cyclizes to form AI-2. Microplate assays revealed no significant difference in biofilm biomass between wild-type and Δ*luxS* mutants, whereas scanning electron microscopy (SEM) demonstrated that Δ*luxS* mutants exhibited looser biofilm architecture, reduced surface coverage, and diminished macrocolony formation capacity. These findings indicate that LuxS primarily influences the physical structure rather than the total biomass of biofilms (Chen et al., 2020).

PNAG (poly-β-1,6-N-acetyl-D-glucosamine), a common bacterial surface polysaccharide, constitutes a crucial EPS component in biofilms. The Type II QS system upregulates the PNAG secretion porin gene *pgaA* in Δ*luxS* mutants via AI-2 signaling, leading to increased PNAG secretion—a mechanism that may enhance bacterial persistence within biofilms (Chen et al., 2020).

In Chen et al.’s study (Chen et al., 2020), the *wzm* gene encoding a membrane protein involved in O-antigen translocation was downregulated in Δ*luxS* mutant biofilm extracts, suggesting LuxS may regulate LPS synthesis during early biofilm formation. Conversely, De Araujo et al. (2010) observed upregulation of the *wzm* gene in *K. pneumoniae* LM21 Δ*luxS* mutant biofilm extracts compared to wild-type extracts. These discrepancies may stem from genetic variations among bacterial isolates or methodological differences in experimental conditions.

### 2.6 Heteroresistance

Heteroresistance in *K. pneumoniae* refers to the phenomenon where a small resistant subpopulation coexists within a predominantly antibiotic-susceptible bacterial population (Stojowska-Swędrzyńska et al., 2021; Lin et al., 2024). This can lead to undetected resistant subpopulations during routine antimicrobial susceptibility testing, resulting in clinical treatment failure and infection recurrence (Wang et al., 2022). The mechanisms are diverse, including: gene dosage effects (e.g., increased *bla_CTX-M-15_* and *bla_SHV33_* β-lactamase gene copy numbers causing heteroresistance to piperacillin-tazobactam) (Babiker et al., 2024; Nicoloff et al., 2024); carbapenem heteroresistance (e.g., upregulated *bla_KPC_* copies and reduced OmpK35/36 expression in CRKP leading to ceftazidime-avibactam resistance) (Li et al., 2024); and polymyxin heteroresistance (prevalence 6.2%-71.9% in CRKP, associated with *phoPQ*/*pmrD* overexpression or *mgrB* mutations) (Weng et al., 2023). This heteroresistance significantly complicates treatment, potentially causing monotherapy failure with carbapenems or polymyxins (Xiong et al., 2021), and may regenerate *in vitro* under antibiotic pressure. Studies suggest combination therapies like ceftazidime-avibactam plus tigecycline or elevated avibactam concentrations (8–16 mg/L), as well as polymyxin-aminoglycoside (e.g., amikacin/gentamicin) or polymyxin-carbapenem (e.g., meropenem/imipenem) combinations can suppress resistant subpopulations (Rajakani et al., 2023). Current research limitations include unstandardized detection methods (e.g., PAP assay) and predominance of *in vitro* models (Abichabki et al., 2025). Future studies should investigate *in vivo* evolutionary processes and links between heteroresistance and persister cells (Li et al., 2023).

## 3 Phage-based therapeutic approaches against *K. pneumoniae*

### 3.1 Phages and their derived enzymes

Phages, as crucial biological factors regulating bacterial populations in nature, offer a novel paradigm for anti-infection therapy through their unique lytic mechanisms. The typical phage infection cycle initiates with the molecular recognition between receptor-binding proteins (RBPs) and specific ligands on the host surface, with this precise targeting determining the phage’s host specificity (Dowah and Clokie, 2018). Taking the T5 phage from the Siphoviridae family as an example, the RBP pb5 at the tip of its central tail fiber specifically recognizes the outer membrane receptor FhuA on Escherichia coli, triggering conformational changes in the tail structure. Subsequently, the hub protein pb3 at the baseplate coordinates with the tail tube protein pb2 for ejection and transmembrane channel formation, ultimately facilitating the injection of phage DNA into the host cytoplasm through the newly established channel (Linares et al., 2023). Based on differences in infection strategies, phages can be classified into two major functional categories: temperate and lytic. The former utilizes integrase to stably incorporate its genome into the host chromosome as a prophage, achieving vertical transmission through bacterial cell division (Howard-Varona et al., 2017); The latter employs the holin-endolysin dual-component lysis system for programmed host lysis—holin proteins form pores in the cell membrane, enabling endolysin to penetrate the peptidoglycan layer, ultimately causing osmotic imbalance-induced lysis (Young, 2014). This precise lytic mechanism endows phage therapy with three core advantages: exceptional target specificity, superior biosafety, and rapid bactericidal kinetics.

**Figure 2:**
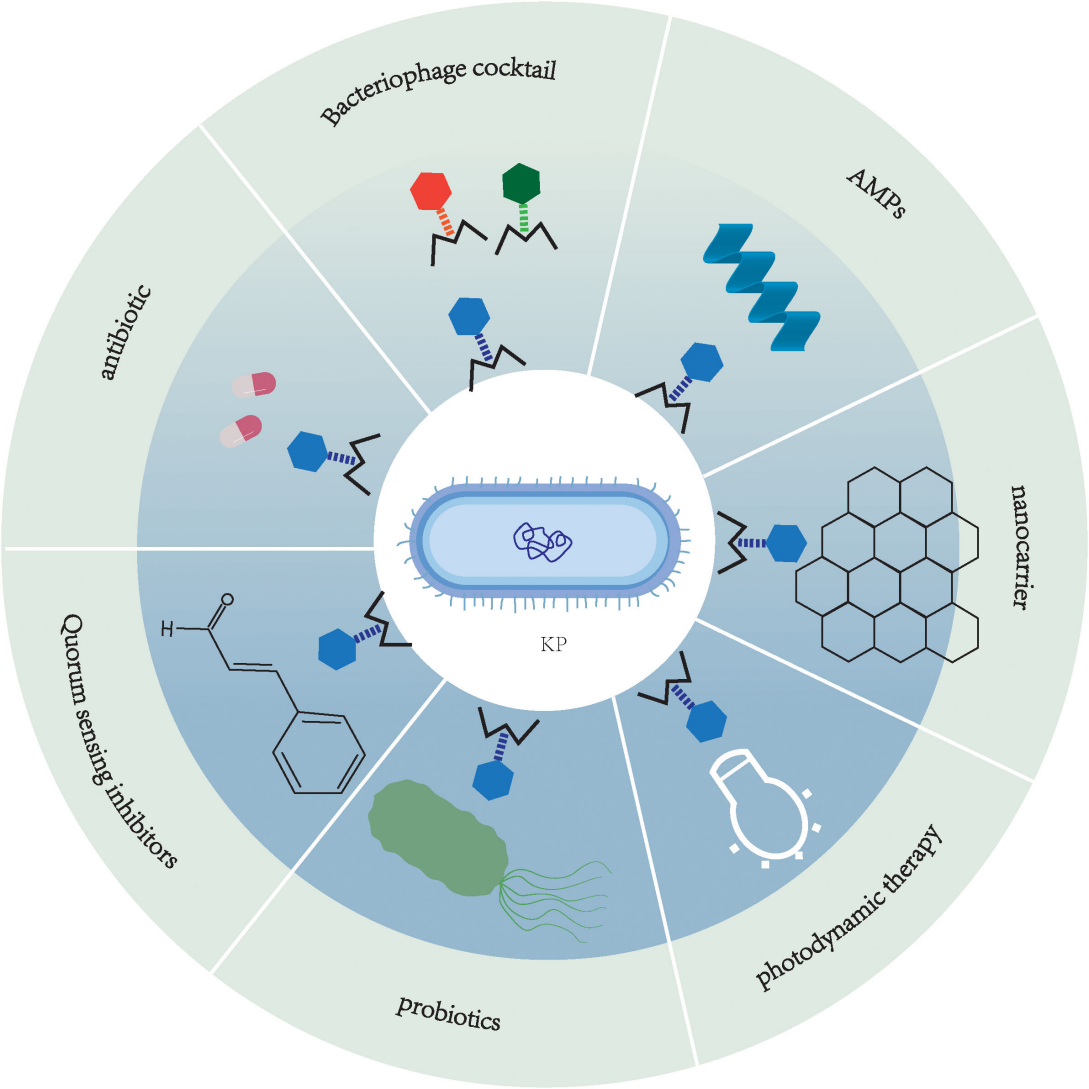
Phage-based combination therapies for the treatment of *K. pneumoniae* Infections.

In phage therapy systems, lyases have garnered significant attention due to their unique molecular advantages. These bacteriophage-encoded peptidoglycan hydrolases achieve bacteriolysis by specifically recognizing and cleaving core chemical bonds in bacterial cell wall peptidoglycan layers. Compared to intact phages, lyases exhibit broader-spectrum activity and are less likely to induce bacterial resistance, with their safety profile having been validated in multiple animal models (Wang et al., 2020; Vasina et al., 2021). Euler et al. (2023) characterized the lyase PlyKp104 derived from a *K. pneumoniae* phage genome. Its transglycosylase domain and C-terminal amphipathic α-helix, with positively charged sequences, synergistically enable specific penetration through the outer membranes of Gram-negative bacteria. *In vitro*, this enzyme demonstrated rapid bactericidal activity (complete sterilization within 10 minutes) against clinical isolates including ESBL and CRKP, while maintaining high efficacy across broad pH ranges (5.0-10.0), high salt conditions (500 mM NaCl), and pulmonary surfactant environments. In murine skin infection models, a single topical application of 300 µg Ply*K. pneumoniae*104 reduced the bacterial load of drug-resistant *K. pneumoniae* by >2 logs. What’s more, it should be noted that prolonged *in vitro* exposure experiments failed to induce resistant mutants, highlighting its promising potential as a novel therapeutic agent against MDR gram-negative bacteria. Moreover, the K1 lyase developed by Tu et al. (2022) features a trimeric β-helical structure containing both catalytic and non-catalytic carbohydrate-binding sites. This enzyme specifically degrades *K. pneumoniae* K1 CPS through β-elimination reactions while preserving critical antigenic epitopes (pyrophosphorylation and O-acetylation modifications). In both *in vitro* phage adsorption assays and high-dose murine infection models, the enzyme demonstrated significant virulence reduction with 100% survival rates. Notably, the 8-day treatment regimen induced neither resistance mutations nor toxic reactions. Despite the remarkable advantages of phage therapy, its standalone application faces multiple challenges. Host bacteria can evade phage attacks through various evolutionary mechanisms, including adaptive mutations such as receptor epitope modifications, CRISPR-Cas system activation, and Abi system expression (Wang et al., 2023). Furthermore, individual variations in phage pharmacokinetic parameters and stringent host specificity limitations significantly constrain the predictability of clinical efficacy (Wunderink, 2019; Pessina et al., 2023). To overcome these bottlenecks, researchers are actively developing multimodal combination therapies: constructing phage cocktail formulations to broaden host coverage (Chen et al., 2024); combining lyases with antibiotics to generate synergistic bactericidal effects (Hong et al., 2023), or integrating nanocarrier technology to achieve targeted delivery and sustained-release enhancement, etc. as shown in **Figure 2** (Kaur et al., 2021). These multidimensional synergistic strategies not only reduce the risk of resistance development but also amplify bactericidal efficacy through coupling different mechanisms of action. Clinical studies employing phage therapy against *K. pneumoniae* are presented in **Table 3**.

**Table 3:** Clinical research summary of phage therapy against *K. pneumoniae*(KP)(2019-2024).

Infection type	Year	*K. pneumoniae*	Resistance	Strategies	Antibiotics	Course	Administration route	Outcomes	Refs
Pulmonary infection	2023	NA	MDRKP	ΦKP_GWPB35(monotherapy) → ΦKP_CWPB35 +ΦKP_CWPA139 (cocktail)	Concomitant Antibiotics	two treatment courses	Aerosolization	Symptomatic improvement occurred without complete pathogen eradication, with phage-resistant bacterial strains emerging that exhibited reduced virulence.	(Li et al., 2023)
UTIs	2023	NA	ESBL	Metamorpho + Mineola + pKP20 (cocktail)	NA	4 weeks	Intravenous injection (IV)	No recurrence within 1 year, with complete eradication of ESBL-producing strains	(Le et al., 2023)
Prosthetic joint infection (PJI)	2022	NA	ESBL	KP1(2 days)+KP2(2 days)+KP1+KP2(cocktail)	Ertapenem	6 days	IA (4 days,IV(2days)	Symptomatic improvement with no recurrence for 14 months and restoration of joint function	(Doub et al., 2022)
UTIs	2021	NA	NA	Customized phage, Intesti phage, SES phage	vaginal suppository containing metronidazole, miconazole, Centella asiatica extract, polymyxin B, and neomycin	Three treatment courses (20 days + 15 days + 1 month)	Oral administration and vaginal suppository	Pathogen not eradicated	(Zaldastanishvili et al., 2021)
Fracture-related infection (FRI)	2021	ST893	PDR-KP	A preadapted phage M1	Meropenem and colistin, followed by CAZ/AVI	5-day phage therapy followed by 3-month antibiotic treatment	Topical and IV administration	The infection was controlled with no recurrence for 3 years, and the patient regained ambulatory capacity.	(Eskenazi et al., 2022)
Recurrent urinary tract infections (rUTIs)	2021	NA	ESBL	Unspecified phage species	Meropenem	29 days	Rectal administration	Ultimately required nephrectomy for definitive treatment, with unclear therapeutic contribution from phage therapy	(Rostkowska et al., 2021)
UTIs	2021	ST15	MDRKP	Monophage ФJD902; Two-phage cocktail ФJD902 and ФJD905; Three-phage cocktail ФJD905, ФJD907, and ФJD908; Four-phage cocktail ФJD902, ФJD905, ФJD908, and ФJD910	The fourth course of phage therapy combined with piperacillin/tazobactam	four treatment courses	Intravesical instillation and renal pelvis perfusion	Infection eradicated with bladder mucosal improvement and no recurrence for 2 months	(Qin et al., 2020)
Pulmonary infection	2020	NA	PDR-KP	Klebsiella phage KPV811+ Klebsiella phage KPV15	Meropenem	4 days	Inhalation and nasogastric tube administration	No K. pneumoniae was detected in bronchoalveolar lavage, while sensitive strains were isolated from stool samples.	(Rubalskii et al., 2020)
UTIs	2020	ST11	XDR-KP	SZ-1,SZ-2,SZ-3,SZ-6 and SZ-8 (cocktail)+ KP165,KP166,KP167,KP158 andKP169(cocktail)+KP152,KP154,KP155,KP164,KP6377 and HD001(cocktail)	Third course combined with trimethoprim-sulfamethoxazole (SMZ-TMP)	5 days (three courses)	Bladder irrigation	Phage-resistant strains emerged during the first two treatment courses. Complete pathogen eradication was achieved after the third course, with no recurrence observed over six months.	(Bao et al., 2020)
MDR bacterial intestinal colonization (MDR-BIC)	2020	ST307	MDRKP	Customized lytic phage (vB_KPnM_GF)	CAZ-AVI	21 days	Oral and intrarectal administration	Complete eradication of gut-colonizing bacteria	(Corbellino et al., 2020)
UTIs	2020	NA	PDR-KP	Four-phage cocktail (phages 117, 135, 178, and GD168) and three-phage cocktail (phages 130, 131, and 909)	Meropenem + Amikacin	Two 5-day treatment courses	Intravesical instillation	XDR-KP was eradicated, with significant improvement in bladder infection.	(YG. et al., 2020)

### 3.2 Phage-antibiotic combination therapy

The synergistic effects between phages and antibiotics extend beyond the classical paradigm where subinhibitory antibiotic concentrations enhance phage lytic efficiency (Comeau et al., 2007), Recent studies have revealed more molecular-level interaction mechanisms. For instance, antibiotics can modify bacterial outer membrane permeability (e.g., polymyxins disrupting LPS structures), which helps promote phage adsorption and enhance intracellular penetration of other antibiotics, forming a “phage-antibiotic penetration enhancement effect” (Wang et al., 2021b). Besides, the evolutionary cost of phage-resistant strains (e.g., virulence gene loss or metabolic dysfunction) can significantly increase bacterial susceptibility to antibiotics, with this “fitness trade-off” being particularly prominent in *K. pneumoniae*. Qin et al. (2024) demonstrated that combined use of *Klebsiella* phage H5 and CAZ induced point mutations in the *wcaJ* gene. While conferring phage resistance, these mutations caused metabolic reprogramming due to CPS synthesis defects, increasing the strain’s CAZ sensitivity by 32-fold - a perfect illustration of the “fitness trade-off” mechanism. What’s noteworthy is that the study also revealed that PAS (phage-antibiotic synergy) alters bacterial metabolic activity and selection pressure, which can influence population evolutionary trajectories to suppress resistance emergence. Recent studies have further proposed engineering phage RBPs as antibiotic-targeted delivery systems, which through specific recognition of bacterial surface receptors, can dramatically enhance antibiotic concentration at infection sites (Zhao et al., 2024).

Biofilm formation constitutes a critical barrier to antibiotic resistance in *K. pneumoniae*, against which phages demonstrate unique advantages. They penetrate biofilms by secreting polysaccharide depolymerases to degrade key matrix components while utilizing tail structures for precise host receptor recognition, thereby overcoming physical barriers. Subsequently, phages diffuse into biofilms either by reducing adsorption rates or via enzyme-generated channels, infecting metabolically active bacteria and releasing progeny through lysis, ultimately achieving layer-by-layer biofilm clearance from surface to depth (Visnapuu et al., 2022). Notably, planktonic bacteria released after phage treatment exhibit significantly enhanced susceptibility to low-dose antibiotics. For example, phage vB_KquU_φKuK6 targeting *K. pneumoniae* reduced host biofilm-forming capacity *in vitro*, and when combined with chloramphenicol or neomycin, decreased antibiotic MICs by approximately 8-fold (Miller et al., 2024).

Clinical successes of phage-antibiotic combination therapy have progressed from case reports to systematic investigations. Bao et al. (2020) applied a phage cocktail (SZ-1/SZ-2/SZ-3/SZ-6/SZ-8) with SMZ-TMP to an ERKP (ST11-type) recurrent UTI patient. Although SMZ-TMP lacked direct antibacterial activity, the combination effectively suppressed phage-resistant mutants, achieving eventual pathogen clearance. In another FRI case, engineered phage topical administration combined with intravenous polymyxin successfully eradicated a two-year persistent XDR infection, with the patient regaining mobility after 3 years (Eskenazi et al., 2022).

Risks and challenges:

Though phage-antibiotic combinations demonstrate synergistic therapeutic potential, they also present multiple risks and challenges. First, antagonism risks exist: certain antibiotics may inhibit phage replication efficiency. For instance, β-lactams (e.g., CAZ) indirectly reduce phage proliferation by suppressing bacterial growth, leading to diminished bactericidal effects during early combination therapy. Nicholls et al. (2023) observed in Pseudomonas aeruginosa that CAZ-phage combinations showed initial antagonism (6 h) before transitioning to synergy by 24 h, indicating time-dependent effects. Second, resistance evolution risks are significant: phages may facilitate antibiotic resistance gene dissemination among bacteria via transduction, particularly when combined with subtherapeutic antibiotic concentrations that may be selected for MDR strains. Leclerc et al.’s (Leclerc et al., 2022) mathematical modeling demonstrated that concurrent antibiotic-phage administration could promote MDR, as antibiotic-induced bacterial growth suppression limits phage replication efficiency, potentially favoring resistance gene transduction. Experimental data revealed delayed bacterial clearance but lower resistance risks when phages were administered after antibiotics.

Furthermore, during the combination therapy against *K. pneumoniae*, the synergistic effect between phages and antibiotics can indeed significantly inhibit the evolutionary dynamics of drug resistance; however, the mechanism underlying this process is complex and has not yet been fully elucidated (Qin et al., 2024; Zhao et al., 2024; El-Din et al., 2025). Multiple studies have confirmed that the synergy between phages and antibiotics reduces the selection pressure for drug-resistant mutations by altering the evolutionary trajectory and metabolic adaptability of bacteria. For instance, phage-antibiotic combinations (e.g., phage H5 and ceftazidime) can inhibit carbohydrate metabolic activity and induce mutations with high fitness costs (such as galU gene mutations), thereby delaying the emergence of drug resistance (Qin et al., 2024; Yu et al., 2025). Nevertheless, the evolution of drug resistance may still occur. For example, bacteria can escape phage attack through mutations in outer membrane proteins (e.g., OmpK35) or phage receptors (e.g., galU), which may be accompanied by cross-resistance or changes in susceptibility (Rocker et al., 2020; Yu et al., 2025). These dynamics are crucial for predicting therapy failure, as differences in evolutionary pathways (such as mutation types and fitness costs) directly affect the sustainability of clinical efficacy (Anastassopoulou et al., 2024).

A third key challenge lies in clinical standardization, including unresolved issues regarding administration timing, dosage optimization, and adjuvant antibiotic requirements. Chan et al. (2018) found that lower antibiotic doses may outperform higher ones when combined with phages, though this depends on specific bacterial species, antibiotics, and phage types. Clinical case studies (Williams et al., 2023)further highlight the absence of unified protocols: among 18 chronic pulmonary patients, 15 required adjuvant antibiotics, yet significant variations existed in administration routes (nebulization, IV, etc.) and treatment durations, with some patients experiencing recurrence post-symptomatic improvement.

Current research focuses on exploring synergistic mechanisms and optimization strategies, yet faces three critical bottlenecks: (1) Lack of universal patterns across bacterial species/antibiotic classes; (2) Unclear evolutionary dynamics of phage-antibiotic interactions during long-term use; (3) Insufficient pharmacokinetic and safety data for clinical translation.

In the post-antibiotic era, the development of phage-antibiotic synergistic therapy regimens is still constrained by multiple barriers, with significant bottlenecks particularly in key aspects such as the standardization of phage preparations, the regulation of host immune responses, and the adaptability of regulatory frameworks. These issues directly impede the translational efficiency of such therapies from the laboratory to clinical practice. On one hand, policy and regulatory constraints, coupled with ambiguities regarding biological risks, constitute fundamental obstacles. The current regulatory system has not yet established a comprehensive, flexible, and sustainable institutional framework. It lacks dedicated approval criteria for the “live biological agent + chemical drug” combination therapy model and fails to form unified evaluation standards for biological risks (e.g., phage-mediated transduction of antibiotic resistance genes, potential integration of phages with the host genome), thus making it difficult to adapt to the patient-oriented clinical application needs of novel phage-based therapies (Pirnay et al., 2022). On the other hand, the lack of standardization for phage preparations is a prominent issue. As live microbial agents, the production of phages is significantly influenced by the activity of host bacteria and fermentation conditions. The titer of phage preparations can fluctuate by up to 10^3 plaque-forming units (PFU)/mL across different batches, and there are no unified control limits for impurities such as endotoxins and host proteins. This inconsistency renders the results of multi-center clinical studies difficult to compare and validate. Concurrently, the interference of host immune responses further exacerbates the uncertainty of therapeutic efficacy. During treatment, patients may generate phage-specific antibodies, which accelerate phage clearance and compromise the synergistic effect (Nang et al., 2023). Additionally, some immunocompromised patients may experience non-specific inflammatory reactions; however, there remains a lack of mature immunomodulatory strategies to balance such risks and therapeutic benefits. Furthermore, large-scale *in vivo* experimental data verifying the efficacy and toxicological properties of phage preparations are still insufficient. Meanwhile, major pharmaceutical companies lack motivation to invest in phage research and development, primarily due to challenges in intellectual property protection for phage technologies and the high costs associated with commercial development and promotion. The interplay of these multiple factors has resulted in significant difficulties in advancing the clinical translation of phage-antibiotic synergistic therapy regimens. Notably, a novel strategy using phage lysins, specific enzymes encoded by phage genomes that exert antibacterial effects through targeted disruption of bacterial cell walls, is emerging. Their chemical nature circumvents risks associated with live virus applications, demonstrating substantial potential in overcoming existing regulatory hurdles and biosafety controversies.

### 3.3 Phage cocktail therapy

Phage cocktail therapy, by combining phages targeting different receptors of the same bacterial species or different species, has emerged as a crucial strategy against drug-resistant *K. pneumoniae* infections through synergistic antimicrobial effects. Its core mechanisms involve: (1) Broader host range coverage reducing resistance escape risks from single-phage receptor mutations; (2) Enhanced bactericidal efficiency via diverse lytic pathways; (3) Rational phage combinations dramatically lowering concurrent multi-phage resistance probabilities by selecting phages with incompatible mutation sites (Rohde et al., 2018; Wunderink, 2019; Wang et al., 2024). An Israeli team’s Cocktail 5 (containing MCoc5c, KP2-5-1, etc.) significantly reduced ST323-type *K. pneumoniae* colonization and inflammation in murine colitis models without inducing resistance (Federici et al., 2022); Meanwhile, NIH researchers successfully suppressed ST258-type CRKP dissemination using Pharr and φKPNIH-2 combinations, with early intervention markedly improving survival despite later resistance recurrence (Hesse et al., 2021). Clinical successes include Le et al.’s (Le et al., 2023)intravenous three-phage regimen (Metamorpho, Mineola, pKP20) eradicating recurrent ESBL *K. pneumoniae* UTIs in a renal transplant recipient with one-year recurrence-free survival.

Risks and challenges:

Phage cocktail therapy, as an emerging antimicrobial strategy, demonstrates significant potential against specific MDR pathogens while facing multiple risks and challenges. Firstly, bacterial heterogeneity and the rapid evolution of phage resistance pose major obstacles. For instance, in treating MDRKP infections, the presence of polyclonal subpopulations in patients led to three consecutive phage treatment failures, with success only achieved through combined phage cocktail and antibiotic therapy (Qin et al., 2020). Secondly, the host specificity of phages limits their broad applicability. A pediatric renal transplant case showed partial efficacy using an Escherichia coli-targeted phage cocktail but required highly personalized phage selection and prolonged treatment duration (Gainey et al., 2023). Moreover, factors like cocktail stability, dosage, and administration timing significantly impact outcomes. In immunocompromised murine models, optimal efficacy required phage cocktail administration within 6 hours post-infection, with delayed treatment markedly increasing mortality (Patel et al., 2021).

Multiple studies indicate that successful phage cocktail therapy depends on precise bacterial matching and optimized combinations. For MDRKP infections, researchers achieved effective control of multifocal infections by screening cocktails against 21 heterogeneous strains (Qin et al., 2020). Similarly, in diabetic mouse models, the AB-SA01 phage cocktail significantly reduced MRSA wound infections, though efficacy relied on phage breadth and dose optimization (Kifelew et al., 2020).

Current research is transitioning phage cocktails from laboratories to clinics, but several critical issues remain. First, phage host specificity and rapid bacterial resistance necessitate the development of broader-spectrum, more stable cocktails. Next, standardization and scalable production represent key future research directions. Besides, machine learning models offer novel approaches for rapid cocktail screening and personalized therapy (Keith et al., 2024).

### 3.4 Phage-antimicrobial peptide combination therapy

Antimicrobial peptides (AMPs), small molecular proteins composed of short-chain amino acids, exhibit broad-spectrum antibacterial activity by disrupting bacterial membrane integrity and serve as key components of innate immunity (Jain et al., 2023). Recently, phage-AMP combination therapy has emerged as a research focus against drug-resistant *K. pneumoniae* infections due to its synergistic potential. The cooperative mechanisms involve: (1) Target complementarity: phage-mediated lysis releases endolysins that cooperate with AMPs to disrupt the cell wall-membrane bilayer, accelerating bacterial disintegration; (2) Enhanced biofilm penetration: AMPs destabilize biofilm matrices, facilitating phage infiltration to deep bacterial layers; (3) Resistance barrier breakthrough: dual-action mechanisms reduce cross-resistance risks through single escape pathways (Mirski et al., 2019; Wang et al., 2021a; Tyagi et al., 2024). Hagh Ranjbar et al. (2023) demonstrated that combining phages with ϵ-polylysine (ϵ-PL) significantly outperformed monotherapies against colistin-resistant *K. pneumoniae*. The authors propose that ϵ-PL’s cationic properties may electrostatically interact with negatively charged phage tail proteins, enhancing membrane penetration, while simultaneously disrupting bacterial membrane potential to facilitate phage DNA injection, collectively boosting bactericidal activity.

Risks and challenges:

Current research indicates that synergistic applications of phages and AMPs predominantly employ engineered fusion proteins rather than co-administration of separate components. Although such designs enhance penetration through gram-negative bacterial outer membranes, their clinical translation faces multiple challenges including stability, toxicity, and immunogenicity concerns (Lopes et al., 2022). What’s more, no studies have systematically investigated the clinical feasibility of combining standalone phages with AMPs, particularly for complex *K. pneumoniae* infection scenarios.

### 3.5 Phage combination therapy with other substances

In recent years, innovative combination strategies of phages with other substances have demonstrated diversified approaches against drug-resistant *K. pneumoniae* infections. These combination therapies significantly enhance antibacterial efficacy through multi-mechanistic synergies, offering novel therapeutic solutions for combating MDR bacterial infections.

#### 3.5.1 Phage-nanocarrier co-delivery: enhanced bioavailability

Nanodelivery systems can address the stability challenges of phages and certain active molecules (e.g., enzymes or small-molecule drugs). For instance, liposomal or polymeric microparticle encapsulation techniques have been employed for co-delivering phages with auxiliary components. Studies demonstrate that nanomicrobial technology combining phages with magnetic nanomaterials (e.g., magnetite) effectively enhances phage targeting and stability while reducing resistance development (Qamar et al., 2022).

#### 3.5.2 Phage-photosensitizer combination: photodynamic synergistic bactericidal effect

Photodynamic therapy (PDT) kills bacteria by activating photosensitizers to generate reactive oxygen species (ROS). Recent studies have combined phages with photosensitizers to establish dual antimicrobial mechanisms: phage-lysed bacteria release photosensitizers that further destroy residual bacteria and biofilms upon light activation. Petrosino et al. (2023) developed an M13 phage-based modular platform that functionalizes phage capsids with Rose Bengal photosensitizer, achieving targeted photodynamic bactericidal effects against gram-negative bacteria.

#### 3.5.3 Phage-probiotic combination: remodeling gut microbiota

The combined application of phages and probiotics demonstrates remarkable potential, particularly in remodeling gut microbiota. This combination therapy not only effectively eliminates pathogenic bacteria but also restores and maintains intestinal microbial balance through probiotic activity, thus providing more comprehensive therapeutic outcomes. Liu et al. (2021a) highlighted that phage-probiotic combinations show promise for intervening in gut dysbiosis, especially in treating *K. pneumoniae-*associated chronic liver diseases. Though current research on phage-probiotic combinations remains in the early stages, their potential for precision therapy and microbiota modulation has attracted significant attention.

#### 3.5.4 Phage-QSIs combination: enhancing antibacterial efficacy by disrupting bacterial communication

QSIs disrupt bacterial communication by suppressing QS systems, therefore inhibiting biofilm formation and reducing virulence factor production (Martínez et al., 2023). When combined with phages, QSIs weaken bacterial defense mechanisms and pathogenicity while phages specifically recognize and lyse bacteria (Xuan et al., 2022). This dual approach synergistically enhances antibacterial efficacy: phages directly reduce bacterial load while QSIs impair collective bacterial defenses. Barrio-Pujante et al. (2024) demonstrated that cinnamaldehyde (CAD) significantly downregulated *IsrB* gene expression to suppress QS, rendering previously phage-resistant *K. pneumoniae* susceptible to phage infection, resulting in increased phage proliferation and reduced bacterial growth.

## 4 Concluding remarks and future perspective

As a paradigm of antimicrobial resistance evolution in Gram-negative bacteria, MDRKP has emerged as a critical threat to global public health systems. Through the acquisition of MGEs carrying β-lactamase genes (e.g., *bla_KPC_*, *bla_NDM_*, and *bla_OXA_*), MDRKP develops MDR phenotypes and XDR evolutionary trends, leading to clinical failure of last-resort antibiotics like carbapenems and significantly increasing treatment failure and mortality rates in critically ill patients. Although significant progress has been made in understanding MDRKP resistance mechanisms, knowledge gaps persist regarding the dynamic evolution of its resistome, particularly in key pathogenic processes such as QS regulation, biofilm formation mechanisms, and metabolic remodeling of persister cells - areas lacking clinically actionable therapeutic targets.

Future clinical optimization of phage therapy requires establishing a multidimensional research framework: At the molecular level, elucidating dynamic phage-bacteria interaction mechanisms through cryo-EM to reveal conformational dynamics of receptor-binding domains and in-depth investigation of phage-host interactions will enhance treatment precision. For addressing narrow host ranges, genetic engineering of phages or developing phage cocktails can significantly expand their host spectrum. What’s noteworthy is that engineered phages can utilize CRISPR-Cas systems to deliver guide RNAs targeting MDR genes in *K. pneumoniae*, employing Cas9 nuclease-mediated precise gene editing to disrupt resistance mechanisms and restore antibiotic susceptibility (Nath et al., 2022). CRISPR-enhanced phage engineering should be advanced to improve *in vivo* stability and replication efficiency. Addressing these priorities will make phage therapy a reliable strategy against MDRKP infections. Regarding long-term *in vivo* efficacy, phage stability and pharmacokinetic properties represent critical challenges. Pharmacokinetically, phages only proliferate when the bacterial density reaches certain thresholds (Nilsson, 2019). Consequently, premature or inappropriate phage administration may lead to immune clearance before proliferation occurs. Optimizing inoculation timing and dosage will be pivotal for phage therapy. Furthermore, the interaction mechanisms between phages and host immune systems remain incompletely understood, necessitating future research to explore how phages interact with immune cells and how these interactions influence therapeutic outcomes. large-scale RCTs for MDRKP-infected populations are urgent to confirm efficacy, safety, and genotype-specific effects.

The core of phage-antibiotic antagonism resides in the incompatibility between the mechanism of action of antibiotics and the replication/bactericidal capacity of bacteriophages. Specifically, protein synthesis inhibitors (e.g., aminoglycosides) are capable of significantly suppressing phage replication (Zuo et al., 2021). Furthermore, the type and dosage of antibiotics, along with host microenvironmental factors (such as serum and urine), can modulate whether the phage-antibiotic interaction manifests as synergism or antagonism (Gu Liu et al., 2020). These complexities pose considerable challenges to the clinical implementation of combined phage-antibiotic therapy, especially in the treatment of multidrug-resistant infections. Thus, additional studies are warranted to elucidate the underlying molecular mechanisms governing these interactions and to optimize the corresponding therapeutic strategies.
